# The impact of website quality on customer satisfaction and eWOM in online purchase intention: The moderating role of gender in risk-taking

**DOI:** 10.3389/fpsyg.2022.945707

**Published:** 2022-08-24

**Authors:** Uzman Saleem, Su yi, Muhammad Bilal, Dan Ioan Topor, Sorinel Căpuṣneanu

**Affiliations:** ^1^School of Economics and Management, Harbin Engineering University, Harbin, China; ^2^School of Economics and Management, Anhui Polytechnic University, Wuhu, China; ^3^Faculty of Economic Sciences, 1 Decembrie 1918 University, Alba Iulia, Romania

**Keywords:** e-commerce, website quality, customer satisfaction, eWOM, Chinese consumers, risk-taking in gender, online purchase intention

## Abstract

Recently, social media marketing has become one of the most significant growth channels for many businesses. However, many companies are still unclear about using social media marketing to their advantage, particularly in an e-commerce environment. In this background, this study examines the effect of website quality, consumer satisfaction, and eWOM on online purchase intention. An online survey was conducted with 789 online Chinese shoppers from four cities—Harbin, Shenyang, Guangzhou, and Shenzhen. Structural equation modeling (SEM) was used to analyze the hypotheses. The findings show that each variable had a high impact on eWOM with website quality (information quality, system quality, and service quality), which in turn positively increased consumer online purchase intentions in China's e-commerce business. Additionally, findings show a significant gender gap in online shopping behavior. This novel research provides several managerial guidelines that support managers in improving their business performance in the e-commerce industry. This research also highlighted some limitations.

## Introduction

The development of the internet and the worldwide web (WWW) are both incredibly significant breakthroughs in the field of information communication technology (ICT), which is critical in e-commerce (Yuan et al., 2021). With widespread internet access, the need for e-commerce services is growing. The worldwide e-commerce sale reached $4.29 trillion in 2020. Business-to-consumers (B2C) e-commerce is the most popular kind of e-commerce globally, which contains e-retailers and e-customers (Chen and Yang, 2021). China has the most excellent internet statistics globally, with 989 million consumers and an internet penetration rate of 70.4% (CNNIC, 2021). B2C refers to buying not just from physical stores, for instance, shopping malls, etc., but also from web-based e-retailers in China such as 淘宝 (Taobao), and 京东 (Jingdong).

As a mirror and terminal in e-commerce, a seller's shopping website registers its presence to all users, showcases its products, and provides relevant detailed information to all consumers (Lowry et al., 2014). With the significant development of e-shopping worldwide, e-retailers must study the determinants of website quality (WQ) toward purchase intentions of B2C e-commerce customers. E-retailers primarily use their websites for transaction processes; it has been recognized that the quality of the website is a chief factor for these retailers' success (Qalati et al., 2021). The e-commerce success model, coined by DeLone and McLean (2003), comprised information quality, system quality, and service quality as antecedents of website effectiveness. Numerous researchers have uncovered the effects of WQ on the satisfaction level of customers (Fang et al., 2011; Zhou and Jia, 2018).

E-commerce has become an excellent platform for electronic word of mouth (eWOM) due to its mobility, ubiquity, and interactivity. Communications are abounding on various social media platforms such as online review sites, blogs, social networking sites (SNS), and online discussion forums (Sohaib et al., 2019). In China, there are multiple features of internet use. Social media, like SNS (e.g., WeChat, QQ, Pengyou, Douban, Sina Weibo, and Renren), have become rapidly well-liked among Chinese internet users. The users of SNS are exceedingly quick to share and discuss product experiences, which generates eWOM. Nowadays, eWOM has become a solid channel for influencing consumers' online buying decision process (Israeli et al., 2019). Most customers trust eWOM, and the information by online consumers is considered most essential and trusted compared to information generated by the company (Hayes et al., 2018). eWOM is a leading predictor of customer online purchase intention (OPI) (Jalilvand and Samiei, 2012; Nuseir, 2019). Bhattacharya et al. (2019) demonstrated that eWOM positively impacted the experience in online shopping, which boosts OPI.

Ultimately, eWOM positively constructs OPI for customers finding reviews relevant to products and services posted through other consumers on social media (Park et al., 2007; See-To and Ho, 2014). However, social media also provides enormous information to its users, which could be perilous and enticing for risk-takers. Truong et al. (2014) demonstrated that risk-takers perpetually participate in diffusing eWOM on social media and try out interesting new things and activities, like innovative products, fashions, and newer versions of items. Particularly, according to scholars, there is a difference between men and women in how they process shopping information available on the internet (Bae and Lee, 2011; Sun et al., 2019). Female customers are more passionate and pay attention to complete details than male customers; they are more practical and concentrate on particular information sections during the buying process. Male customers are willing to take risks more based on their experiences. On the other hand, female customers are risk-averse and depend on eWOM (Chu and Choi, 2011; Fan and Miao, 2012). The differences in risk-taking between the genders are a significant factor in the shopping environment that remains neglected and needs study. This can impact the customers' OPI from an e-commerce perspective.

Different studies have been conducted to examine the factors that influence consumers' purchase decisions online. For example, some studies investigate how WQ, customer satisfaction (CS), and eWOM affect consumer emotions leading to online shopping using environment psychology (Chevalier and Mayzlin, 2006; Fan et al., 2017; Aggarwal and Aakash, 2020). These studies indicate that online information, wisdom, and integration of the shopping process will improve online purchasing (Verhagen and Van Dolen, 2011; Wells et al., 2011). Accordingly, we believe that information quality, system quality, and service quality will significantly influence online purchases. A retailer's website is more likely to receive positive eWOM publicity from consumers if its quality is superior (Oh et al., 2015; Bilal et al., 2021a). Consumers are often highly motivated to share positive eWOM about products and an online retailer if their online appearance is attractive, the information is of high quality, and the search capabilities are robust and accessible. Furthermore, advanced WQ usually leads to higher satisfaction ratings (Hsiao et al., 2010). It is highly essential in our context because, while building CS is a complex process, it also plays a prominent role in making consumer purchasing decisions in developed nations such as China, where most consumers are risk-averse (Al-Debei et al., 2015).

The study provides empirical validation of the factors that impact consumers' behavior when shopping online. We investigate how WQ (information quality, system quality, and service quality), eWOM, and satisfaction affect OPI. We also identify the mediating role of satisfaction between WQ and eWOM. Finally, we also explore the moderating role between eWOM and OPI. Consequently, this study is set up to examine the following research questions:

**RQ1**. Does WQ (information quality, system quality, and service quality) influence Chinese consumers' purchase intention online?
**RQ2**. Does satisfaction mediate the relationship between WQ and eWOM?
**RQ3**. Do risk-taking differences between the genders moderate the relationships between eWOM and OPI in the Chinese online environment?

## Literature review and hypotheses development

### Online purchase intention (OPI)

Online purchase intention refers to the customers' decision-making process while buying from an online shopping website after assessing every element they feel is relevant (Hsu et al., 2012). It is complex to measure the purchase intention of an individual (Zhang et al., 2014), but many factors affect OPI. Farzin and Fattahi (2018) stated that purchase intention is the intention of a customer to buy a certain product, service, or brand. OPI is formed even under the assumption of delayed transactions and, as a result, is often regarded as an essential indicator of actual purchase (Chang and Wildt, 1994). The purchase intention has also been considered the main accurate predictor of actual purchase behavior because this is an important stage of actual purchase (Reimer and Benkenstein, 2018; Bilal et al., 2022), benefiting companies.

Moreover, a purchase benefits the company's income and profit directly. Hence, the importance of customers' purchase intentions as an upshot variable of online shopping is explicit in this research. Consequently, customer OPI is a primary endogenous variable in our research design.

### Website quality (WQ)

In e-commerce, the website is where transactions between customers and e-retailers commonly happen (Liang et al., 2011). Some online retailers' websites are more persuasive than others due to their effective WQ features (Shang and Bao, 2020). Aladwani and Palvia (2002, p. 469) define customers' perception of WQ as “consumer's assessments about website's characteristics that meet consumers' needs and represent the cumulative website effectiveness”. The shopping website is not solely a system of information but also an interface between e-retailers and customers (Gefen et al., 2003). Alshibly and Chiong (2015) proposed that for an e-commerce company to succeed and improve its position online and to understand the competition and improve industry benchmarks, it was critical to evaluate the quality of its website.

From an e-commerce perspective, WQ is regarded as a significant internal element for customers to examine the criteria of e-retailers (Kim and Lennon, 2013). Aggarwal and Aakash (2018) highlighted that online shopping websites which deliver better functionality, accessibility, reliability, usability, flexibility, and stability to online consumers could be conceded as a website of high quality. Information Systems (IS) success model, coined by DeLone and McLean (2003), directly evaluates the characteristics of a WQ in e-commerce. They point to three vastly acknowledged dimensions: information quality, system quality, and service quality. The basic factors in assessing expectations and perceptions of website users about WQ could be these three dimensions (DeLone and McLean, 2003; Liang and Chen, 2009). In online CS, these three dimensions of quality play a crucial role (DeLone and McLean, 2003). Information quality is about comprehensive content on the web that enables consumers to perceive provided information. This web content is related, compact, personalized, secure, and understandable for consumers (Chi, 2018). The system quality alludes to the efficiency and features of systems on the web, which are accessible, adaptable, responsive, and reliable to consumers (Chi, 2018). Additionally, service quality refers to the online assistance provided to consumers by the management of the website, as customer service for improving the browsing and shopping experience of consumers (Chi, 2018).

According to Lowry et al. (2014), WQ can be considered the features of a website that helps achieve online CS. It is also a crucial element for retailers' success as the website is an initial impression on every consumer (Akram et al., 2018). Previous scholars propounded that WQ directly impacts CS (Tandon et al., 2020). A high-quality website can maximize reach and satisfaction and generate positive eWOM. Many scholars have found that WQ and CS are directly and positively correlated with each other (Lin, 2007; Shin et al., 2013; Zhou and Jia, 2018). Customers who perceive and experience better quality of a specific shopping website will create eWOM by sharing their experience with relatives, friends, colleagues, and others. Tarkang et al. (2022) revealed that WQ is crucially responsible for eWOM; for instance, when a website user gets a website that is user-friendly, attractive, and easily accessible, there would be a higher probability of the user recommending the website. Based on the above discussion, we hypothesize that:

H1. WQ positively influences CS.
H2. WQ has a positive effect on eWOM.

### Customer satisfaction (CS)

Customer satisfaction refers to the process of comparison of customer expectancy with real product performance after purchase. Hidayat et al. (2016) described CS as a valuation of perceived variance between before purchase expectations about a product/service and the actual performance of the products/services after its usage. Therefore, CS increases when real features meet or exceed anticipations and dissatisfaction increases when real features fail to meet anticipations (Brilliant and Achyar, 2021). From an e-commerce perspective, customers' satisfaction speaks for the overall impression of a website's features or performance (Lin, 2007; Hardiyanto and Firdaus, 2021). CS of a website means an assessment concerning the website features and user experience. Oliver (2010) defined CS as “an assessment based on his/her personal experience relevant to his/her needs and expectations.”

Regarding the elements that influence eWOM (Tsao and Hsieh, 2012; Prayag et al., 2017), stated that CS is positively correlated with the desire of consumers to review and recommend service providers. Lii and Lee (2012) pointed out that companies tend to hope that satisfied consumers will engage in eWOM automatically. From an online shopping perspective, eWOM happens when consumers are either dissatisfied or satisfied with the consumption of the products or services.

Moreover, CS as a vital antecedent of eWOM was proved by Serra-Cantallops et al. (2018) in their research. Besides the direct impact of WQ on eWOM, WQ can influence eWOM also through CS. The authors stated that CS is the mediating factor in the connection between WQ and eWOM because lack of CS can be the basic cause of customers deciding not to provide positive eWOM. Furthermore, some researchers found a direct association between WQ and CS (Zhou and Jia, 2018; Giao et al., 2020). Rizal et al. (2018) highlighted the duality in CS's contribution to the current knowledge and argued that CS was a predictor as well as a mediator of eWOM behavioral intentions. According to the link in relationships stated above, there is a high possibility that CS would mediate the relationships between WQ and eWOM. Therefore, we hypothesized that:

H3. CS refers to the connection of mediation between WQ and eWOM.

### Electronic word-of-mouth (EWOM)

Up to now, numerous authors have defined word-of-mouth (WOM). According to Silverman (2011), WOM is defined as “the exchange of information about products or services among two or more persons, which can be informal and non-commercial communication.” WOM is a crucial ingredient in changing customer behavior about purchase intention. eWOM is a modified version of internet-based communication of WOM in the new technological epoch (Yang, 2017; Bilal et al., 2020). Litvin et al. (2018) defined eWOM as “every informal communication addressed to customers and related to features or usage of products or/and services or e-retailers that is based on the internet.” eWOM is the sharing of personal experiences with specific product/service providers via opinions, online reviews, and suggestions, resulting in persuasive effects on targeted customers (Shin et al., 2014). The continuous progress of Internet technology has boosted the number of consumers who utilize the Internet to find information related to a product or company through eWOM. Consumers not merely read and use related reviews on the shopping websites for their process of purchase decision but also share opinions (eWOM) found on the shopping websites with others (Chua and Banerjee, 2016; Aggarwal and Aakash, 2020). From a WOM perspective, although people essentially share the product experience with their family, friends, and others, at the same time they are also engaged in eWOM on the e-commerce website and social media. In addition, eWOM has a conspicuous benefit because it is available to everybody who can use internet-based channels (i.e., SNS) to share their suggestions and reviews with other consumers.

According to Filieri (2015), online comments and SNS allow consumers to virtually work together and disseminate reviews, information, and feelings about products, services, and brands. Social media has become the most popular platform for eWOM, which is used to swap knowledge about products and services on a daily basis among customers. eWOM has become more trustworthy, valuable, authentic, and enjoyable through these popular platforms for customer OPI (Erkan and Evans, 2014). According to Kietzmann and Canhoto (2013), eWOM can be acknowledged as a free advertisement that supports brands and boosts the sale of the company's products by improving buying rates. At that time, the role of eWOM has become very significant in obtaining information for customers' decision making process (Hussain et al., 2017). An important conclusion from this line of research is that these eWOM contents have a very significant effect on customer OPI (Filieri et al., 2018; Bilal et al., 2021b). Some studies have demonstrated that eWOM significantly influences customers' OPI. Tarkang et al. (2022), in their specific study on the airline industry websites, uncovered that, more directly, a significant and positive association exists between eWOM and customers' OPI. Therefore, we hypothesized that:

H4. eWOM positively affects customers' OPI.

### The moderating role of risk-taking in gender

In the last few years, the differences in their propensity for risk-taking among genders on shopping websites has become a critical field of research. Risk-taking (Büttner and Göritz, 2008) is stated as customers' assessment and embracing of possible undesirable results associated with their decision-making process. Pires et al. (2004) described that taking chances and motivation to try out new products/services is relevant. According to Clark and Goldsmith (2005), risk-taking customers are unlike other kinds of persuasive customers, for example, opinion leaders, innovators, and market mavens. Innovative customers are generally ready for the early adoption of products compared to other customers, and opinion leaders influence other people. Market mavens are persuasive due to their familiarity with a brand, including experience related to product or service and market information. These three kinds of persuasive customers also exhibit risk-taking traits at varying levels. For instance, according to Rogers (2010), innovative customers can be exposed to the possibility of high risk due to their proclivity to embrace a new product or service.

Scholars found that risk-taking customers are exposed to new concepts on shopping websites; risk-taking individuals like to accept and respond positively to information willingly from unknown sources (Clark and Goldsmith, 2005; Truong et al., 2014). Moreover, risk-taking customers on shopping websites and social media are active in expressing their opinion in favor or against others' views. Risk-takers are excited to look for fascinating products that are available on shopping websites and to try out novel products, for instance, innovative products, fashionable, a new version of the product, etc. On shopping websites, risk includes various forms of risk such as payment fraud, product quality, psychological, and source. Featherman and Pavlou (2003) defined perceived risk as “any possible loss in the acquisition of expected consequence of the use of e-services”. The researchers estimated that perceived risk is the degree to which an individual is concerned about the possibility of uncertain consequences affecting the customers' decision-making process (Hussain et al., 2017). A lot of investigations revealed that in an online shopping environment, perceived risk is higher than offline or store-based buying environment (Mortimer et al., 2016; Kumar and Bajaj, 2019). The information available on social media and shopping websites can be abundant as well as risky and provides an exciting environment for risk-takers.

The propensity within genders to perceive and use available information on shopping websites differs significantly (Bae and Lee, 2011; Choi and Kim, 2014). According to Putrevu (2001), women pay attention to all information available on shopping websites and compare similarities and disparities from fragmented information. While purchasing online from shopping websites, they are more anxious about risks (Garbarino and Strahilevitz, 2004). Female customers perceive more risk than male customers, leading them to seek more comprehensive information (Meyers-Levy and Loken, 2015; Madan and Yadav, 2018); they are more likely to search all available information in an effortful manner, evaluate information, and post comments in an intensive way for others (Sun et al., 2019). On the contrary, male customers pay attention to a tiny piece of detail, and reviews are regarded as effective for making a decision (Putrevu, 2001). From the social media perspective, previous studies have flagged the gender disparities in risk-taking (Shi et al., 2016). Male customers take more risks while shopping online, take risks (Fan and Miao, 2012), and are more confident than females (Carlin et al., 2018). Hence, according to Kim et al. (2011), female customers are more influenced by eWOM as compared to male customers when shopping online. From a social media perspective, many studies have explored the effect of a person's gender and risk-taking as a moderating factor vis-a-vis different variables (Fan and Miao, 2012; Lim et al., 2017).

Based on the above discussion, we hypothesize that:

H5a. High risk-taking male customers moderate the relationship between eWOM and OPI in e-commerce or online shopping websites.
H5b. High risk-taking female customers moderate the relationship between eWOM and OPI in e-commerce or online shopping websites.

### Methodology

#### Data collection and sampling procedure

This research model was constructed to understand the influence of WQ on CS and eWOM directly and through mediating the role of CS; furthermore, the effect of eWOM on OPI through moderating the role of risk-taking by men and women on Chinese consumers from an e-commerce perspective. When the discernment of the research problem exists, this kind of research usually uses a design that is quantitative and descriptive; the case of the present study is similar. The data was collected online from four cities (Harbin, Shenyang, Guangzhou, and Shenzhen) in China for the survey. Harbin and Shenyang are located geographically in the northeast of China and these cities are sub-provincial and capital cities of their provinces. In contrast, Guangzhou and Shenzhen are located geographically in the southeast of China. Guangzhou is sub-provincial and the capital city of its province, while Shenzhen is a special economic zone. From the perspective of social, economic, cultural, and customer behavior toward e-shopping, northeast and southeast China are different. This research collected study data from Chinese consumers linked with e-shopping. The convenience sampling technique was used for this survey, and questionnaires were distributed online through social-network applications, like WeChat, QQ, and Weibo, which are most popular in China. A web-based survey has some advantages (e.g., broad coverage of geographical response, rapid response, and less costly) and has been extensively used in past studies (Akram et al., 2018; Tariq et al., 2019). The Chinese customers of e-commerce who were targeted for this survey were those who purchased products online through e-commerce apps, such as 淘宝 (Taobao) and 京东 (Jingdong). Hence, the first question was: “Have you bought any product by e-commerce application within the past four months?” If the response was “no”, the questionnaire weblink was disabled.

The survey questionnaire was initially constructed in English. For extreme consideration, the questionnaire was translated into Chinese for Chinese respondents and back-translated into English with the assistance of four language experts from the languages department and two doctoral students from the marketing department who are proficient in English and Chinese. The survey questionnaire consisted of two portions: (a) respondents' personal information, including gender, age, education level, and experience of online purchasing through shopping websites/apps; (b) Items of measurement. Prior to the start of the final survey, the piloted test was performed on the questionnaire through 44 students who were well associated with shopping through e-commerce applications/websites to ensure constructs and scale items' clarity. The piloted test showed that all measurement items were well communicated and understood.

In total, 850 responses were collected from targeted Chinese customers in three months from March-May 2021. Forty-one responses which were incomplete and twenty invalid responses were removed from the data set. Finally, 789 responses were considered for the final data analysis of this study. Few gifts were offered to the respondents by researchers through a lucky draw. The respondents were 18 years or older; most were between 18 and 27 years. The majority of respondents were college students (37.64%) and read online reviews (the most crucial factor) (89.23%). The largest group of respondents (68.82%) had been involved in online shopping for over two years. Table 1 shows the participants' demographic information.

**Table 1:** Demographics of research sample (*N* = 789).

**Measure**	**Group**	**Frequency**	**Percentage**
Age	<18 years	37	4.69%
	18–22	223	28.26%
	23–27	196	24.84%
	28–32	111	14.07%
	33–37	129	16.35%
	<37	93	11.79%
Gender	Male	473	59.95%
	Female	316	40.05%
Education	High school or below	23	2.92%
	College	297	37.64%
	Bachelors	271	34.35%
	Masters or above	198	25.09%
Visiting SNS	Yes	789	100.00%
	No	0	0.00%
Reading online reviews	Yes	704	89.23%
	No	85	10.77%
Frequency online reviews	>1 years	285	36.12%
	1–2 year	211	26.74%
	3–4 year	196	24.84%
	<4 year	97	12.30%
Posting online comments	Yes	595	75.41%
	No	194	24.59%
Online shopping experience through WeChat.	>1 year	94	11.91%
	1–2 year	305	38.66%
	3–4 year	277	35.11%
	<4 years	113	14.32%

### Instruments

All measurement items were extracted from reliable instruments found in previous studies (Cronbach's alpha 0.70). Specifically, WQ, four items of measurement for each relating to information quality, system quality, and service quality, was adopted by Zhou (2012). Four items scale were measured for satisfaction adopted from Fang et al. (2011) and Shin et al. (2013). eWOM was measured through six items by Cheung and Lee (2012). A three-item scale was used to measure purchase intention by Chen and Barnes (2007). Further, the three items scale was measured for moderating variables of risk-taking in gender adopted from Taylor et al. (2011). A five-point Likert scale was used (1 = “strongly disagree”; 5 = “strongly agree”) to measure each item.

#### Reliability and validity

For the data entry and analysis, we employed statistical packages for the social science (SPSS) 22.0 and (AMOS) 22.0, which were created by IBM (USA). Based on possible interactions/relations between WQ (information quality, system quality, and service quality), CS, eWOM, and risk-taking in gender with the mediation of satisfaction and moderation of risk-taking in gender variables and their effects on customer's online purchase intention in e-commerce perspective, measures validation was confirmed through SEM which was used in two steps: confirmatory factor analysis (CFA) and hypotheses testing. First, AMOS 22.0 was implemented to perform CFA to evaluate the factor structure and the model fit of the latent variables. Next, the structural model was scrutinized to analyze the hypothesized relationships among exogenous and endogenous variables.

The CFA retained items when they loaded over 0.50 in one factor (Arnold and Reynolds, 2003) and less than 0.30 in the other factor (Klein et al., 1998). The measuring model showed fit of the model, Table 2 (chi-square = 357.61, df = 2.63, RMSEA = 0.7, RMR = 0.07, GFI = 0.93, AGFI = 0.90, NFI=0.97). The constructs were reliable, with (a) coefficient alpha estimates ranging from 0.79 to 0.98, well above the 0.70 threshold (Hair et al., 1998), and composite reliability (CR) estimates increasing the 0.70 threshold in all constructs and (c) average variance extracted (AVE) greater than the recommended 0.50 threshold (Nunnally and Bernstein, 1978; Fornell and Larcker, 1981; Hair et al., 1998). Table 3 displays the results.

**Table 2:** Results of model fit.

**Fit**	**CMIN/df**	***P*** **-value**	**RMSEA**	**CFI**	**GFI**	**AGFI**	**NFI**	**RMR**
Recommend value	<3	<0.05	<0.08	>0.90	>0.90	>0.80	>0.90	<0.08
Measurement model	2.63	0.005	0.7	0.94	0.93	0.90.	0.97	0.7
Structural model	2.58	0.005	0.6	0.93	0.92	0.92	0.98	0.06

**Table 3:** Reliability and convergent validity.

**Constructs**	**Items**	**Means**	**SD**	**Item loading**	**CR**	**AVE**	**Cronbach's**
Information quality	4	3.132	1.45	0.79–0.87	0.879	0.727	0.878
System quality	4	3.265	1.57	0.82–0.85	0.897	0.798	0.978
Service quality	4	3.101	1.34	0.76–0.83	0.818	0.717	0.798
Customer satisfaction	4	4.415	1.32	0.78–0.88	0.89	0.737	0.865
eWOM	6	3.897	1.42	0.83–0.91	0.867	0.709	0.924
Purchase intention	3	3.564	1.47	0.81–0.93	0.851	0.767	0.989
Risk taking	3	4.654	1.39	0.84–0.94	0.869	0.787	0.861

*SD, standard deviation; CR, composite reliability; AVE, average variance extracted*.

The maximum likelihood factor loadings, which were both high and significant, indicated convergent validity for the underlying constructs (Wixom and Watson, 2001). That is, all factor loadings were greater than 0.5 and highly significant. Discriminant validity implies that each latent variable represents a distinct construct (Scott and Scott, 1998). This was determined by comparing a factor's item loadings to its cross-loading on other factors. Each factor loading was greater than the cross-loadings on unplanned factors (Henseler and Chin, 2010a,b). Discriminant validity was also verified because the correlations between the factors were less than the square root of the AVEs, as shown by the bolded values on the diagonal (Fornell and Larcker, 1981) (see Table 4).

**Table 4:** Discriminant validity.

**Constructs**	**1**	**2**	**3**	**4**	**5**	**6**	**7**	**Eigen values**	**% of variance**	**Cumulative** **%**
(1) Information quality	**0.798**							1.99	26.47	26.47
(2) System quality	0.235^***^	**0.879**						2.20	18.20	44.67
(3) Service quality	0.313^***^	0.2251^**^	**0.849**					2.54	16.19	60.86
(4) Satisfaction	0.197^**^	0.312^***^	0.313^***^	**0.797**				2.67	13.12	73.98
(5) Electronic word-of-mouth	0.311^***^	0.421^***^	0.567^**^	0.512^**^	**0.868**			1.89	11.12	85.1
(6) Purchase intention	0.411^**^	0.502^**^	0.334^***^	0.442^***^	0.312^***^	**0.889**		2.65	9.14	94.24
(7) Risk taking	0.421^**^	0.467^***^	0.457^***^	0.567^***^	0.499^**^	0.497^***^	**0.891**	1.79	5.76	100

## Results of proposed hypotheses

Structural equation modeling (SEM) was used after the CFA analysis to analyze the measuring model and test the hypotheses proposed. The structural model test resulted in a good fit between the model and the data (CMIN/df = 2180.43, df, 895, *p* < 0.01, GFI = 0.90, CFI = 0.95, TLI = 0.92, RMSEA = 0.05, and RMR = 0.05). All hypotheses were supported except H5b. For H1, WQ significantly influenced satisfaction with a coefficient of 0.76, *p* < 0.001. Similarly, for H2, WQ significantly influenced eWOM with a coefficient of 0.57, *p* < 0.001. In H4, eWOM positively influenced purchase intention with coefficient of 0.47, *p* < 0.05. Hence, H1, H2, and H4 were supported (Table 5).

**Table 5:** SEM result.

***H***	**Relationship**	**Estimates**	**SE**	**CR**
**H1**	Website quality→ Satisfaction	0.76	0.163	2.412^***^
**H2**	Website quality→ eWOM	0.57	0.178	2.332^***^
**H4**	eWOM→ Purchase intention	0.47	0.261	2.169^**^

*** *p < 0.001,*

** *p < 0.05*.

Hypothesis 5a investigated the moderating effects of risk in gender (i.e., male, female). To assess the effects of the moderator, hierarchical regression analysis was used. First, an analysis was done to investigate the linear and moderating impact of eWOM on male risk-taking. The following variables were added to the model in the following order: eWOM, risk-taking, eWOM × risk-taking. In the first and second steps, the main effect of eWOM (β= 1.221) remained significant. The main effect of risk-taking was found in males; however, a significant interaction between eWOM and risk-taking (β = 0.689) on customer purchase intention was discovered from an e-commerce perspective—male customers' high risk-taking levels moderate the relationship between e-WOM and OPI. As a result, H5a was supported (Table 6).

**Table 6:** The moderating effect of risk-taking in males.

	**β**	***t*** **-value**	***F***	***R*** **^2^**	**Adjusted *R*^2^**	**Δ*R*^2^**
eWOM	1.221^***^	8.991	394.134^***^	0.672	0.657	0.648^***^
RT	0.687^**^	3.912	201.789^***^	0.678	0.589	0.017^**^
eWOM × RT	0.689^*^	3.567	150.345^***^	0.681	0.623	0.211^*^

*F statistics are used for overall models. eWOM stands for electronic word of mouth, RT stands for risk-taking, and the dependent variable is purchase intention*.

* *p 0.05,*

** *p 0.01 and*

*** *p 0.001*.

Second, an analysis was carried out to determine the linear and moderating effects of eWOM on risk-taking in females. The following variables were entered into the model in the following manner: eWOM, risk-taking, eWOM × risk-taking. In the first and second steps, the main effect of eWOM (β= 1.623) increased significantly. The main effect of risk-taking in females and the interaction effect of eWOM and risk-taking (β= −0.022) on customer purchase intention from an e-commerce perspective were not reported. Female customers' high risk-taking levels were not moderated in the relationship between eWOM and OPI. As a result, H5b was not supported (Table 7).

**Table 7:** The moderating effect of risk-taking in females.

	**β**	***t*** **-value**	***F***	***R*** **^2^**	**Adjusted *R*^2^**	**Δ*R*^2^**
eWOM	1.623^***^	−4.623	338.224^***^	0.587	0.667	0.651^***^
RT	−0.383^**^	−1.432	241.181^***^	0.798	0.689	0.079^**^
eWOM × RT	−0.022^*^	−0.331	161.061^***^	0.771	0.718	0.001^*^

*F statistics are used for overall models. eWOM stands for electronic word of mouth, RT stands for risk-taking, and the dependent variable is purchase intention*.

* *p 0.05,*

** *p 0.01 and*

*** *p 0.001*.

Due to criticism in several studies, the indirect test methods of Hayes and Preacher (2014) were used instead of Baron and Kenny (1986a,b) “the causal step approach” to test the mediating roles of satisfaction. This process does not account for the mediator's indirect effect on the ultimate prospect of making an estimated error. Furthermore, the confidence interval for the mediation effect is not given. Because of the aforementioned reasons, Bootstrap was used (Preacher and Hayes, 2008). The indirect effect testing syntax developed by Preacher and Hayes into an SPSS processor was used to run tests with all significant mediators in one model. The data were bootstrapped 5000 times, and the results showed that satisfaction (*z* = 1.1565, *p*< *0.05*) mediates the relationship between WQ and eWOM at a 95% confidence interval for H3 (Table 8).

**Table 8:** Results of bootstrap analysis.

**Variables**		**Indirect effects of proposed mediators**			**Bootstrapped**	
		**Product of coefficient**			**Confidence intervals**	
	**Effect**	**SE**	**Z**	**Boots SE**	**Lower**	**Upper**
**Website quality and e-WOM**						
Satisfaction	0.048^*^	0.105	1.1535	0.0169	0.0233	0.0491

*Level of confidence interval: 95.00, Bootstrapped at 5,000;*

* *p < 0.05*.

## Discussion

Retaining customers, facilitating their services, and providing them quality goods and services are important tasks for an e-retailer for long-term success. The main purpose of this study was to create a robust understanding of the dimensions of WQ (i.e., information quality, system quality, and service quality) and their relationship with CS and eWOM directly and with the mediation of satisfaction. Furthermore, the study sought to explore the eWOM relationship with OPI through moderation of risk-taking in gender (i.e., male, female). Therefore, a cohesive model was proposed for customers' online purchase intention from the perspective of e-shopping/e-commerce in China. The results of this study indicate that the relationships among all variables were supported (directly and indirectly), and the constructed research model was accepted. More directly, H1 predicted that a significant and positive connection exists between WQ and CS, and H2 predicted a substantial and positive association between WQ and eWOM. These relationships established that WQ is considerably important for CS and eWOM; for example, when a consumer perceives the quality of the website to be higher, more attractive, and user-friendly, there is a high probability of CS and positive eWOM for this website. Hypothesis 3 predicted that a positive association exists between CS and eWOM. Relatively, the relationship between WQ and eWOM was fully mediated by CS (Table 8). This research offers support for the contested duality of CS being both a mediator and predictor of eWOM behavior. Our research shows that satisfied customers are most likely to contribute to eWOM compared to dissatisfied customers.

Moreover, H4 indicated that eWOM affects customer OPI available on social media and e-commerce websites (online reviews). Our results showed that effective eWOM remarkably influenced customers' decision-making process and enormously increased the OPI toward buying a product or service. Past research alluded to the positive effect of eWOM on purchase intentions based on social media platforms. According to Balakrishnan et al. (2014), marketing literature point out that eWOM is an important factor in promoting purchase intentions. Particularly, eWOM actively shapes the desires of potential customers regarding the process of decision-making related to buying on shopping websites (King et al., 2014). Currently, Chinese social media users are influenced by it and often read online reviews carefully to estimate products/services, which helps them decide to purchase (Dellarocas, 2006; Akram et al., 2018). The growing importance of online reviews motivates marketers to disseminate positive information by effectual EWOM relevant to products/services; this positively enhance customers' OPI.

Hypothesis 5a highlighted the moderating impact of risk-taking in male customers between eWOM and OPI relationship. Our results revealed that high levels of risk-taking by male customers positively and significantly moderates the relationship between eWOM and OPI. The results confirmed that male customers have strong OPI because they are risk-takers. Fan and Miao (2012) expostulated that male customers tend to take more risks in online shopping websites due to a lack of attention to detailed information. Notably, Truong et al. (2014) stated that risk-takers are attracted to new things and excited to try out novel products and services available on e-commerce websites. Hypothesis 5b proposes the moderating impact of risk-taking propensity in females between eWOM and OPI relationship. The findings revealed that high risk-taking by female customers did not significantly attenuate the connection between eWOM and OPI. This indicated that female customers lack strong OPI because they are risk-averse. Female customers take less risk because they rely more on reviews and eWOM, (Garbarino and Strahilevitz, 2004; Kempf and Palan, 2006), and to a great extent evaluate the reviews exhaustively and deal with information more intentionally. Specifically, Hupfer and Detlor (2006) stated that female customers are well connected socially and believe in eWOM and friends' suggestions for shopping websites. This research found that male customers believed more in risk-taking and heuristic-technique than female customers from the e-commerce perspective. Female customers trusted online reviews and recommendations more than male customers. Male customers pay less attention to eWOM and take risks to try new or innovative products and services available on shopping websites. Conversely, female customers pay more attention to detailed information associated with products and services to decrease the risk associated with online shopping.

**Figure 1:**
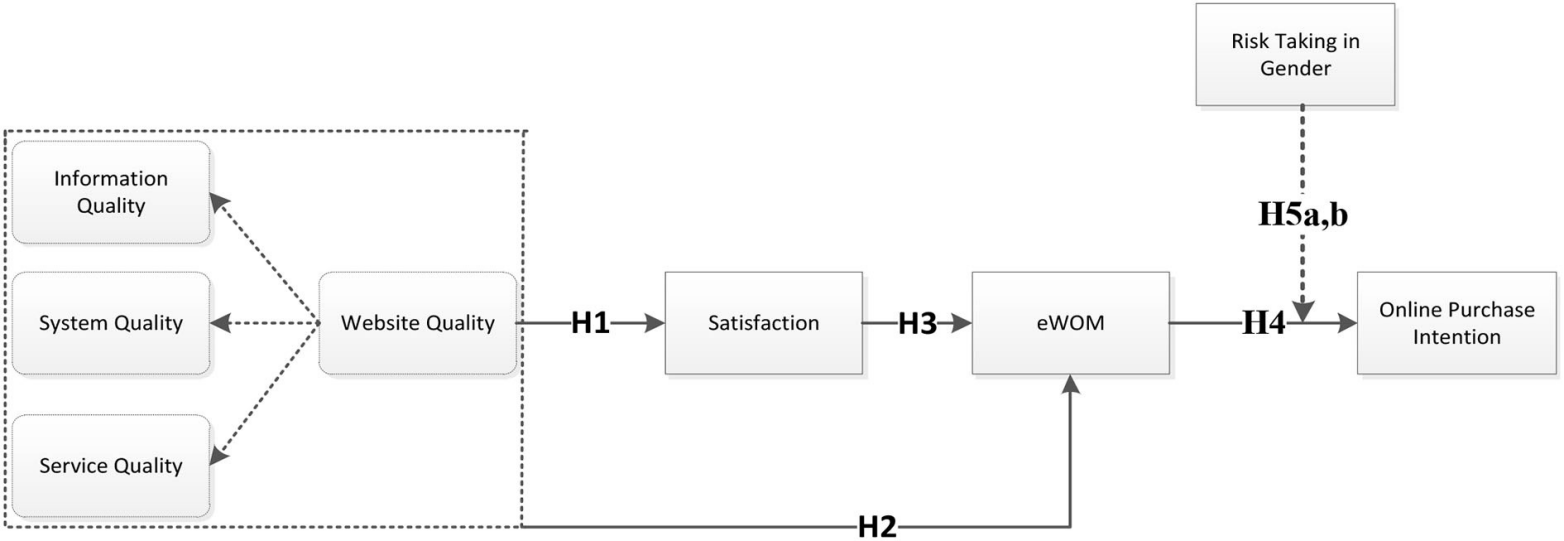
Conceptual framework.

The research results almost fully support the proposed model shown in Figure 1. The study uncovered the importance of WQ dimensions (i.e., information quality, system quality, and service quality) for CS and eWOM toward customer behavior of OPI with the mediation of CS and moderation of risk-taking in gender, especially in the context of e-commerce. Erkan and Evans (2016) demonstrated the direct impact of eWOM on OPI, especially on shopping websites and social media perspectives. In addition, this research examined whether and how risk-taking between the two genders interacts with e-WOM and influences OPI. Risk-taking propensity in male customers positively and significantly moderated the association between e-WOM and OPI. E-retailers on shopping websites and social media could benefit by tailoring their marketing strategies based on the tendency of risk-taking in gender. Furthermore, e-retailers can create online groups to encourage the shopping behaviors of risk-averse customers by generating and providing positive e-WOM regarding products and services. Particularly, it would provide assistance to female shoppers as they pay more attention to detailed information and tend to socialize more with their friends on social media than their male counterparts.

## Theoretical implication

This research makes several contributions to marketing literature. First, this research sheds some light on the salience of WQ from the perspective of B2C e-commerce and its impact on CS and eWOM (directly and indirectly) in China. However, WQ has been widely investigated, such as WQ toward customer's e-loyalty (Giao et al., 2020), and relationship quality with customers through WQ (Zhou and Jia, 2018). Recent research highlighted the importance of WQ for the airline industry and the purchase intention of customers (Tarkang et al., 2022). Second, this study relates to eWOM and customer behavior of OPI in Chinese online shopping websites, i.e., Taobao and Jingdong, and social media such as WeChat and Weibo. Since online shopping website reviews and social media is immensely powerful in determining the possible impact of EWOM on customer OPI, e-retailers can prepare and induce persuasive eWOM related to products or/and services, which may affect OPI from a shopping website's perspective. Specifically, the inclination of risk-taking in gender as a function of moderation was not examined in previous studies in the context of e-commerce, which this study has contributed to. Third, in this study, risk-taking in gender played a vital role in the relationship between eWOM and OPI, and satisfaction mediated the relationship between WQ and eWOM. The empirical results of this study verified the moderating impact of risk-taking in male customers while shopping online.

Conversely, the moderating impact of risk-taking in female customers was not found. According to Fan and Miao (2012), female customers are risk-averse and depend significantly on e-WOM. Finally, this study unfolds a new research approach exploring how WQ affects CS and eWOM toward OPI from the perspective of B2C Chinese shopping websites. This study also guides the mediation of CS and moderation of risk-taking in gender. It sheds light on existing literature relevant to B2C e-commerce social commerce and e-marketing.

## Managerial implication

This study has made an essential contribution to research on online shopping. The results of this study provide specific substantial implications for e-retailers, managers, and marketers who prepare strategic plans and implement tools to improve their e-commerce performance as well.

First, this research could assist e-retailers in understanding the significant factors that entirely determine the customers' OPI, which could help them in improving their IT and managerial strategies and escalate profits. This outcome shows the significance of WQ, CS, and positive eWOM in forecasting the OPI for e-retailers in China.

Second, e-retailers must invest in good quality websites to retain existing and attract potential customers. The importance of WQ could be acknowledged by its positive and significant effect on CS and eWOM of Chinese e-commerce consumers. This finding sheds light on information quality, system quality, and service quality and provides an insightful implication for providing users with a better e-shopping experience. Managers should commit to improving the operating system better and making the website quick and easily accessible. User-friendly and high-quality e-commerce websites have accurate product information, applicable and reliable system, and perfect customer service (active customer support, delivery service, and secure payment mechanism). Chinese consumers are more likely to prefer this kind of website when they intend to order online products.

Third, this research indicates that CS is a mediator and predictor of eWOM. Satisfied customers are like ambassadors for retailers' websites. If customers are not happy with the online experience, they will stop using it, no matter how useful or well designed. On the contrary, if customers feel satisfied, they will repurchase products or services and spread positive eWOM. Therefore, to retain existing and attract potential customers, e-retailers should pay more attention to making customers feel satisfied with the quality of the products, services, and website.

Fourth, this study's results offer online retailers and social media marketers practical insights for creating better discernment relevant to the eWOM impact on customers' OPI from a B2C e-commerce perspective. WeChat and Weibo have become the most potent marketing tools for spreading eWOM on Chinese social media. Consumers on these platforms share their personal experiences of the products and reviews with other consumers on social media. Marketers need to understand how to be present and monitor these platforms of social groups. Additionally, marketers should focus more on delivering consistently reliable and updated information to relevant social media groups. They should enhance their marketing strategies by recognizing the customers' participation in eWOM communications based on shopping websites and social media.

Finally, exploring the moderating impact of risk-taking tendency in gender was exciting. Marketers should formulate different strategies recognizing the variations in risk-taking tendencies of genders. Male customers are more willing to take risks and try out new things available on shopping websites, while female customers are less willing to take risks and depend on online reviews/comments before purchasing the products on shopping websites. Online retailers can use these insights to improve product sales on shopping websites. This study helps local and global marketers by offering a better understanding of the Chinese internet shopping environment. E-retailers can embrace the opportunity by learning everything there is to know about eWOM in China.

## Limitations and future research

This study extended certain important insights relating to customers' OPI into online shopping research. However, this study has different recommendations and limitations for future research. The data collection was among participants recruited from four Chinese cities (Harbin, Shenyang, Guangzhou, and Shenzhen). Hence, survey results primarily reflect customers' online purchase intentions in China. Surveys in other cities could offer further crucial insights. Future research could concentrate on other cities in the four regions of China or replicate it in other countries to obtain international insights.

Second, website quality is a crucial concept from an e-commerce perspective and is expansive and multidimensional. This research explored three dimensions/facets: information quality, system quality, and service quality to measure WQ. Nonetheless, WQ still has many competitive concepts and dimensions. Future research could explore these. Respondents answered questionnaires based on various websites rather than answering questions concerning any particular website. Specific websites can influence customers' OPI and experience of online shopping. This study used risk-taking in gender as moderating factor; future research could use moderating effects such as e-trust.

## Data availability statement

The original contributions presented in the study are included in the article/supplementary material, further inquiries can be directed to the corresponding author/s.

## Ethics statement

Ethical review and approval was not required for the study on human participants in accordance with the local legislation and institutional requirements. Written informed consent from the [patients/participants OR patients/participants legal guardian/next of kin] was not required to participate in this study in accordance with the national legislation and the institutional requirements.

## Author contributions

All authors listed have made a substantial, direct, and intellectual contribution to the work and approved it for publication.

## Conflict of interest

The authors declare that the research was conducted in the absence of any commercial or financial relationships that could be construed as a potential conflict of interest.

## Publisher's note

All claims expressed in this article are solely those of the authors and do not necessarily represent those of their affiliated organizations, or those of the publisher, the editors and the reviewers. Any product that may be evaluated in this article, or claim that may be made by its manufacturer, is not guaranteed or endorsed by the publisher.
